# Biocontrol of Soft Rot Caused by *Pectobacterium odoriferum* with Bacteriophage phiPccP-1 in Kimchi Cabbage

**DOI:** 10.3390/microorganisms9040779

**Published:** 2021-04-08

**Authors:** Soohong Lee, Nguyen-Trung Vu, Eom-Ji Oh, Aryan Rahimi-Midani, Thuong-Nguyen Thi, Yu-Rim Song, In-Sun Hwang, Tae-Jin Choi, Chang-Sik Oh

**Affiliations:** 1 Department of Horticultural Biotechnology, Kyung Hee University, Yongin 17104, Korea; tnghd0708@naver.com (S.L.); nguyen12sh@gmail.com (N.-T.V.); djawl0189@naver.com (E.-J.O.); ntthuong.vnua@gmail.com (T.-N.T.); yulimy@khu.ac.kr (Y.-R.S.); hongkong10@hanmail.net (I.-S.H.); 2 Department of Microbiology, Pukyong National University, Busan 48513, Korea; aryan_rahimi2011@yahoo.com (A.R.-M.); choitj@pknu.ac.kr (T.-J.C.); 3 Graduate School of Biotechnology, Kyung Hee University, Yongin 17104, Korea

**Keywords:** bacteriophage, *Pectobacterium*, phiPccP1, soft rot

## Abstract

*Pectobacterium odoriferum* has recently emerged as a widely infective and destructive pathogen causing soft-rot disease in various vegetables. Bacteriophage phiPccP-1 isolated from Pyeongchang, South Korea, showed lytic activity against *P. odoriferum* Pco14 and two other *Pectobacterium* species. The transmission electron microscopy and genome phylograms revealed that phiPccP-1 belongs to the *Unyawovirus* genus, *Studiervirinae* subfamily of the *Autographivirinae* family. Genome comparison showed that its 40,487 bp double-stranded DNA genome shares significant similarity with *Pectobacterium* phage DU_PP_II with the identity reaching 98% of the genome. The phiPccP-1 application significantly inhibited the development of soft-rot disease in the mature leaves of the harvested Kimchi cabbage up to 48 h after Pco14 inoculation compared to the untreated leaves, suggesting that phiPccP-1 can protect Kimchi cabbage from soft-rot disease after harvest. Remarkably, bioassays with phiPccP-1 in Kimchi cabbage seedlings grown in the growth chamber successfully demonstrated its prophylactic and therapeutic potential in the control of bacterial soft-rot disease in Kimchi cabbage. These results indicate that bacteriophage phiPccP-1 can be used as a potential biological agent for controlling soft rot disease in Kimchi cabbage.

## 1. Introduction

Bacterial soft-rot disease caused by *Pectobacterium* species has been considered as an affliction of vegetables [1]. This genus is currently classified into 18 species: *P. carotovorum, P. atrosepticum, P. aroidearym, P. aquaticum, P. betavasculorum, P. cacticidum, P. fontis, P. parmentieri, P. polonicum, P. Polaris, P. peruviense, P. Punijabense, P. wasabiae, P. zantedeschiae, P. versatile, P. odoriferum, P. brasiliense, P. actibidiae* [2,3,4,5,6,7,8,9,10,11,12,13,14]. Importantly, *P. carotovorum* and *P. atrosepticum* have been considered among the top 10 economically and scientifically important bacterial plant pathogens [15]. They commonly cause serious soft-rot diseases in many important crops including Kimchi cabbage, due to the high secretion level of cell-wall degrading enzymes [16]. In the field, the infection initially occurs on the petioles of bottom leaves and rapidly spreads into the main stems causing the entire plant to collapse within a few days [17]. Moreover, soft rot is not only found in the field, but also in distribution and storage or during marketing, resulting in great economic losses [18]. 

*P. odoriferum* has been generally considered to have a narrow host range and isolated most often from chicory [19,20]. However, it has emerged to cause soft-rot disease on a wide range of storage vegetables [21,22]. In addition, the soft-rot symptoms and tissue maceration caused by *P. odoriferum* are more severe than those caused by *P. carotovorum* in potato [21,23].

In general, chemical bactericides such as antibiotics and copper-based compounds have been extensively used to combat most bacterial plant diseases, including bacterial soft rot. Although these compounds were shown to be effective, they negatively affect the environment and are not effective any more if bacterial strains are resistant to antibiotics or copper-derived compounds [24,25]. So, the development of eco-friendly approaches to manage plant bacterial diseases seems to be relevant. Crop rotation with other non-host crops has been used for control of bacterial soft rot [26]. In addition, biological approaches based on bacteriocin [27,28] or antagonists [29,30] have been demonstrated to effectively control soft rot. 

In recent years, bacteriophages specifically infecting and killing bacteria have emerged as a promising approach for control of bacterial plant diseases [31]. Several bacteriophages to potentially control soft-rot disease caused by *P. carotovorum* in several important plants have been reported, such as My1 [32], PP1 [33], PM2 [34], PPWS1 [35], PP2 [36], PP16 [37], and POP72 [38]. However, no bacteriophages were isolated or demonstrated to effectively control soft-rot disease caused by *P. odoriferum*. In addition, bacteriophage-based biocontrol for soft-rot management in Kimchi cabbage has not been applied yet.

In this study, plant residues of Kimchi cabbage were collected in Pyeongchang of South Korea and used for isolating bacteriophages against *P. odoriferum*. The potential of the isolated bacteriophage, phiPccP-1, as a biocontrol agent for soft-rot disease caused by the target bacterium was evaluated in the mature leaves after harvest and seedlings of Kimchi cabbage grown in the growth chamber.

## 2. Materials and Methods

### 2.1. Bacterial Strain Identification and Growth Conditions

All bacterial strains used in this study are listed in Table S1. For identification of *Pectobacterium* strains, a single colony of each bacterial strain was used as a template for PCR using PrimeSTAR^®^ HS DNA Polymerase (TAKARA Bio Inc, Otsu, Japan) to amplify partial 16S rRNA gene region with universal 16S rRNA bacterial primer set, 27mF (5′-AGAGTTGATCMTGGCTCAG-3′) and 1492mR (5′-GGYTACCTTGTTACGACTT-3′) [39]. PCR products were cloned into pTOP Blunt V2 vector (Enzynomics, Daejeon, Korea). Two clones per each strain were analyzed for identification by sequencing. 

Bacterial strains of *Pectobacterium* spp. were cultured in LB media (10 g L^−1^ of tryptone, 5 g L^−1^ of yeast extract, 10 g L^−1^ of NaCl, pH 7.2). *Dickeya* spp. were cultured in TSB (Tryptone 15 g L^−1^, Soytone 5 g L^−1^, NaCl 5 g L^−1^) or King’s B media (per L of distilled H_2_O: proteose peptone No. 3 20 g, glycerol 10 mL, K_2_HPO_4_ 1.5g, MgSO_4_ 1.5 g. All bacterial cultures were incubated at 26 °C for 18 h. 

### 2.2. Bacteriophage Purification and Propagation

Kimchi cabbage residues were collected from various fields in Pyeongchang, South Korea, where soft-rot disease has occurred. Rotted plant tissues were collected and homogenized in distilled water by shaking for 30 min. After centrifugation (8000× *g*, 10 min, 4 °C) and filtration through a 0.22 μm pore-size filter (Sartorius AG, Göttingen, Germany), the supernatant was subjected to overlay assay with each strain of the *P. odoriferum* strains listed in Table S1, as described previously [40]. Briefly, 100 μL of supernatant were added into 5 mL of soft LB medium (0.4% agar) inoculated with 100 μL of bacterial suspension (OD_600_ = 0.3 to 0.5) and then poured onto solid LB plates. After 30 min of solidification, the plates were incubated overnight at 26 °C. Bacteriophage was semi-purified using five-time repeated overlay assays against susceptible bacterial strains by picking individual plaques and eluting in sodium chloride-magnesium sulfate (SM) buffer (50 mM Tris-HCl (pH 7.5), 100 mM NaCl, 10 mM MgSO_4_). 

*P. odoriferum* Pco14 was employed to propagate the bacteriophage phiPccP-1. Small-scale phage propagation required 5 mL of fresh Pco14-LB cultured (10^8^ CFU mL^−1^) inoculated with 100 µL filtered-plaque suspension in a shaking incubator at 26 °C and 140 rpm. After 16 h of incubation, the supernatant as phage lysate was collected by centrifugation at 10,000× *g,* 4 °C for 10 mins and then was filtered with a 0.22 µm pore-size filter. The large-scale propagation also was conducted as described above with a targeted multiplicity of infection (MOI) of 1. For purification, phage particles were precipitated by treatment with 10% polyethylene glycol (PEG) 6000 (Sigma-Aldrich, St. Louis, MO, USA) and 1 M NaCl [41] and purified using ultracentrifugation (37,000× *g*, 2 h, 4 °C) combined with CsCl density gradient of 1.25, 1.37, and 1.50 g mL^−1^. After dialysis with cold SM buffer at 4 °C, the purified phages were stored in glass tubes at 4 °C.

### 2.3. Transmission Electron Microscopy

The purified phage phiPccP-1 (approximately 1 × 10^9^ PFU mL^−1^) was absorbed onto carbon-coated copper grids for 2 min, and the buffer solution was removed by Whatman paper. Then, the samples on the grids were negatively stained with 2% aqueous uranyl acetate (UrAc, pH 4.0) for 1 min, and the extra UrAc solution was blotted off. The phages were observed by transmission electron microscope (TEM; Tecnai^TM^ G2 Spirit TWIN at 120 kV, FEI Co., Hillsboro, OR, USA) at the Korea Basic Science Institute. Phage classification was performed based on the guidelines of the International Committee on Taxonomy of Viruses [42].

### 2.4. Determination of Host Range of phiPccP-1

The lytic activity of phage phiPccP-1 against *Pectobacterium* strains listed in Table S1 was determined by the spot assay. Briefly, 5 mL of soft medium with targeted bacteria (0.4% agar) inoculated with 100 μL of the overnight culture suspension were poured onto the surface of the same solid medium. After 30 min of solidification, a single drop of phage lysate (approximately 10^8^ PFU mL^−1^) was spotted onto inoculated LB plates, which were incubated overnight at 26 °C. The occurrence of lysed areas on the spot inoculation were observed to indicate lysis efficacy. The experiment was repeated three times independently for verification.

### 2.5. Determination of Killing Curves of phiPccP-1

To evaluate the activity of phiPccP-1 in bacterial growth inhibition, the *P. odoriferum* Pco14 and *P. carotovorum* Pcc15 were employed for the killing curve assay at different MOIs (MOI 10, MOI 1, and MOI 0.1). Briefly, the overnight bacterial culture was harvested by centrifugation and resuspended in fresh LB medium to reach 0.2 of the optical density at 600 nm (OD_600_ = 0.2; approximately 10^8^ CFU mL^−1^). Then, 900 μL of bacterial suspension was loaded into SPL 24-well flat-bottomed plates, and 100 μL of each phage dilution in SM buffer were aliquoted to the corresponding well to reach the targeted MOI. Sterile SM buffer was used instead of phage dilution as a negative control, and LB broth was used as a blank. Each MOI was replicated three to five times. The bacterial growth was monitored by OD_600_ every 15 min using the SPECTROstar^®^ Nano (BMG LABTECH, Ortenberg, Germany), with incubation at 26 °C on a rotary shaker at a rate of 100 rpm. The killing curves were verified twice independently with similar results.

### 2.6. The Frequency Measurement of Bacteriophage-Insensitive Mutant (BIM) Development

To examine the phage-resistant mutant development of *Pco*14 strain, the spontaneous *Pco*14 mutants were isolated, as previously described in Frampton et al. [43], with some modification. Briefly, the 100 μL overnight culture of Pco14 was inoculated with 100 μL phage titer at MOI 1 using the plaque assay. The plates were incubated at 26 °C for over 48 h to fully develop BIMs. The emerging colonies were measured for determination of the frequency of BIMs (number of surviving colonies/original bacterial titer). This assay was repeated twice independently with similar results. Further, four single colonies of each replication were selected by streaking at least 3 times to remove any phage particle and then re-tested against phiPccP-1 infection using the spotting assay.

### 2.7. Thermal, pH, and UV Radiation Stability of phiPccP-1

The phage stability under various environmental conditions such as temperature, pH, UV radiation were carried out in vitro three times independently. The phiPccP-1 phage suspension (10^9^ PFU ml^−1^) was treated at diverse temperatures (0 °C, 26 °C, 45 °C, and 54 °C) for three weeks for thermal stability. The pH stability was tested with 10 μL phage suspension in 990 μL SM buffer solutions adjusted to pH 2, 3, 4, 5, 7, 10, 11, and 12 overnight. Stability of phage under UV radiation was tested according to a previous report [44] with a modification. Briefly, 1 mL of phage lysate (10^8^ PFU ml^−1^) was added into the 24-well plate and then irradiated for 0, 15, 30, 45 and 60 min with the UV light (λ = 365 nm; 320 mW/m^2^). The survival and lytic activity of phages were determined by spotting 10 μL of serially diluted (10 folds) phage lysates on the plate lawn with Pco14 strain. 

### 2.8. Genome Analysis of phiPccP-1 Genomic DNA

The 10^9^ PFU ml^−1^ of fresh phiPccP-1 phage lysate was concentrated by PEG treatment, and then a mixture of DNases and RNase was treated for 30 min at 37 °C right before genomic DNA (gDNA) extraction using the Promega wizard DNA clean-up kit (Promega catalog #A7280, Madison, WI, USA). The purified gDNA was sequenced by Macrogen Inc. (Seoul, Korea) using the PacBio RS II method as well as a HiSeq2500 platform (Illumina). The sequencing library was prepared using the SMRTbell template preparation kit for PacBio RS II and TruSeq Nano DNA kit for Illumina following the manufacturer’s protocol. After subreads filtering of raw data, the subreads were de novo assembled using CANU v. 1.7 assembler [45] and then refined with Pilon v. 1.21 [46]. Then, the annotation of assembled genome was performed by Prokka v. 1.13 [47]. ORFs were predicted using GeneMarkS (v. 4.28) [48] and annotated by alignment against the sequences from the NCBI non-redundant database as well as using InterProScan 5 [49]. The genome information was deposited to the GenBank database (GenBank accession number: MW001769). Genome comparison was conducted with BLASTN and visualized with Easyfig [50]. The phylogenetic tree was constructed by MEGAX [51], using the neighbor-joining method with the Poisson correction model. One thousand bootstrap repetitions were performed. 

### 2.9. Control Efficacy of phiPccP-1 in the Detached Mature Leaves of Kimchi Cabbage

The phages (10^8^ PFU mL^−1^) resuspended with 10 mM MgCl_2_ and also supplemented with 0.02% silwet (Momentive, Waterford, NY, USA) were sprayed on the surface of the detached mature leaves from the harvested Kimchi cabbage until the leaf surface was completely wet. After two h of drying, the leaves were wounded with a sterile scalpel dipped in bacterial suspension of Pco14 strain (10^8^ CFU mL^−1^) for inoculation. Agrimycin®, a commercial antibiotic-based product (Pfizer, New York, NY, USA), and 10 mM MgCl_2_ were used as positive and negative controls, respectively. Inoculated leaves were incubated in the closed plastic boxes at 26 °C. This assay was repeated three times independently with similar results.

### 2.10. The Prophylactic and Therapeutic Effects of phiPccP-1 in Kimchi Cabbage Seedlings

Three-week-old Kimchi cabbage seedlings (*Brassica campestris* L.ssp, *Perkinensis Rupr.* cv. ‘Bom No Rang’, Asia Seed Co., Seoul, Korea) grown in the growth chamber (26 °C, 68–70% humidity, 16 h:8 h light–dark cycle) were used for all assays. The phage phiPccP-1 (10^9^ to 10^10^ PFU mL^−1^) was concentrated using PEG and homogenized in 10 mM MgCl_2_ buffer before treatment. The seedlings were treated with Pco14 strain (approximately 10^8^ CFU mL^−1^). Both bacteriophages and pathogens were sprayed in 30 seedlings with different order, depending on the experiment purposes. Agrimycin^®^ and 10 mM MgCl_2_ buffer were used as positive and negative controls, respectively. For prophylactic efficiency of phiPccP-1, the phages were treated at 2 h before bacterial application, while, for its therapeutic efficiency, it was applied at 6 h after bacterial treatment. The control efficacy of phages in soft-rot disease management was tested with two concentrations at MOI of 0.1 and 1. All treated seedlings were covered by clear plastic covers for 18 h after the first treatment. The disease progress was monitored at 16, 24, and 48 h after pathogen treatment. Both assays were repeated three times independently with similar results.

### 2.11. Statistical Analysis

To statistically analyze disease severity results, the nonparametric Kruskal–Wallis test and Tukey test (*p* < 0.05) were employed. For other results, Duncan’s multiple range test (*p* < 0.05) was performed with SAS (v. 9.4 for Windows; SAS Institute, Cary, NC, USA). 

## 3. Results

### 3.1. Isolation and Classification of phiPccP-1

**Figure 1:**
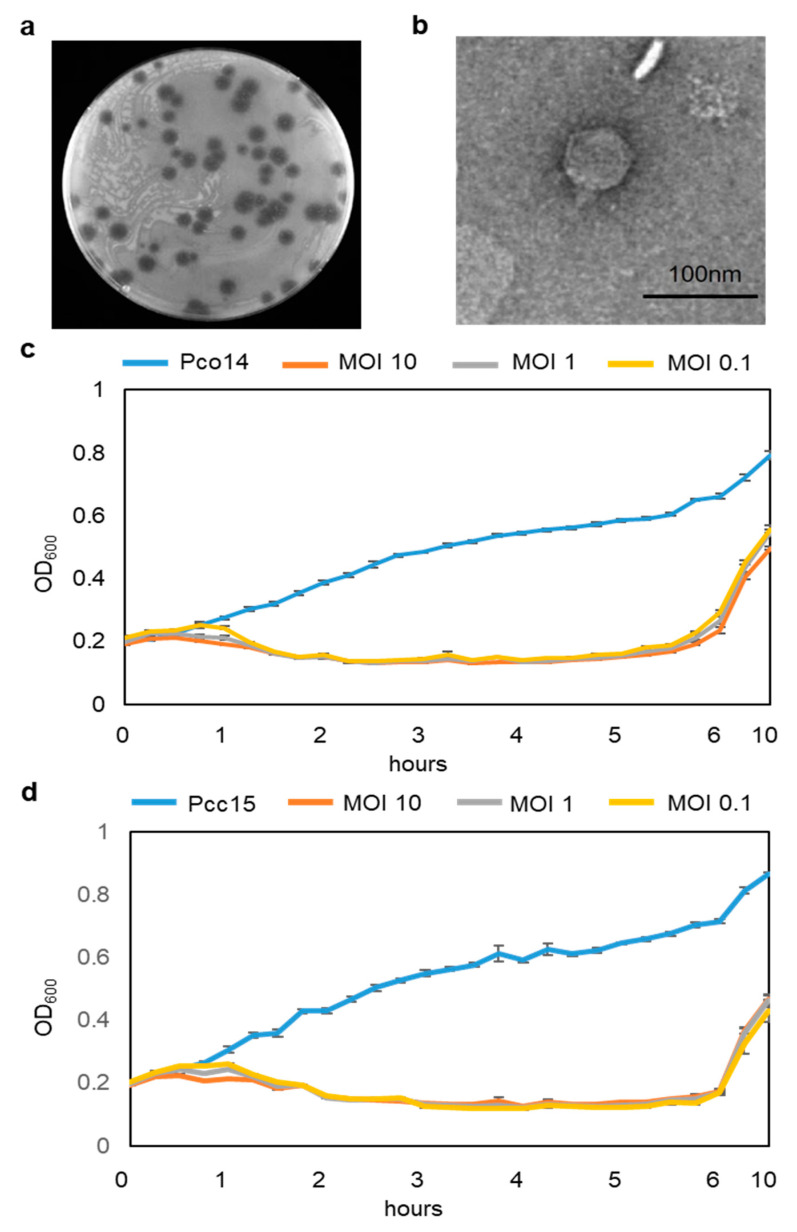
Morphology and in vitro killing curve displaying the activity of phiPccP-1. (**a**) Plaque morphology of the bacteriophage phiPccP-1 against *Pectobacterium odoriferum* Pco14. (**b**) Virion morphology of phiPccP-1 observed by transmission electron microscope. (**c**,**d**) Killing curves against *P. odoriferum* Pco14 (**c**) and *P. carotovorum* Pcc15 (**d**). Bacterial concentration was measured via optical density at 600 nm (OD_600_) every 15 min. Value is average of five replicates, and error bars indicate standard deviation.

Various bacteriophages were isolated from rotted Kimchi cabbage leaves collected from Pyeongchang, South Korea. Among them, phage phiPccP-1 formed clear and large plaques, 3–6 mm in diameter on the bacterial lawn of *P*. *odoriferum* Pco14 (Pco14) (Figure 1a). Therefore, it was chosen for further experiments. TEM analysis revealed that phiPccP-1 phage possessed an icosahedral head (approximately 60 nm in diameter) and an approximately 20 nm short, non-contractile tail (Figure 1b).

### 3.2. Determination of phiPccP-1 Host Range and its Killing Curve

To determine the antibacterial activity of phiPccP-1, spot-on-lawn assays with serially diluted bacteriophage solutions on agar plates were performed against a collection of 39 bacterial strains including 8 of *P*. *odoriferum* isolates, 18 of other species of *Pectobacterium* including *P. carotovorum, P. brasiliense, P. parmentieri, P. atrosepticum, P. betavasculorum, P. wasabiae* and *P. versatile* and 4 strains of *Dickeya* spp. (Table S1). The phage specifically infected and killed 18 of *Pectobacterium* strains causing soft rot in the diverse vegetables including 6 out of 8 tested *P. odoriferum* strains, 5 of 6 tested *P*. *brasiliense* strains, and 6 of 12 tested *P. carotovorum* strains. No lytic activity against other *Pectobacterium* spp. and *Dickeya* spp. was observed. These results indicate that phiPccP-1 bacteriophage has potential to control not only *P. odoriferum*, but also *P. carotovorum* and *P*. *brasiliense*.

To examine the dynamic of phiPccP-1 against *Pectobacterium* strains, *P. odoriferum* Pco14 and *P. carotovorum* Pcc15 were selected and employed for killing curves assay. PhiPccP-1 could suppress the growth of both strains at all MOIs of 10, 1, and 0.1 within the first 5 or 6 h (Figure 1c,d). Bacterial optical density continuously increased from 0.2 to over 0.8 in bacteria alone treatment, while the co-culturing of target bacteria with bacteriophages made optical density decrease to 0.1 within the first 2 h. Although there was no difference in phiPccP-1 dynamic in case of different host strains, the different MOIs led to the difference of starting decrease time points. Indeed, the bacterial density started to reduce after 30 min at the MOI of 10, while the reduction was observed after 30 and 45 min at the MOI of 1 and 0.1. The development of insensitive bacteria was observed at around 5 and 6 h after inoculation in Pco14 (Figure 1c) and Pcc15 (Figure 1d), respectively. These results indicate that phiPccP-1 bacteriophage can kill both *P. odoriferum* and *P. carotovorum* efficiently at the early time points and the resistant mutant strains might occur as time goes on. 

### 3.3. The Frequency of BIM Development 

Bacteria develop various mechanisms to overcome the bacteriophage infection [31]. Therefore, the development of bacteriophage-insensitive mutants is a considerable factor for biocontrol with bacteriophages. The spontaneous mutant development of *P. odoriferum* Pco14 against PhiPccP-1 infection was monitored using the plaque assay at the MOI of 1. The number of Pco14-BIM cells was 2866.6 ± 188.5 cells/plate. The monitored frequency of BIMs was 7 × 10^−6^. Further, four emerging colonies were randomly selected and purified to examine whether the wild-type phage was able to lyse its corresponding BIMs. The results showed that phiPccP-1 infected and killed those selected BIMs with lower efficiency, compared to the wild-type Pco14 (Figure S1).

### 3.4. Stability of phiPccP-1 under Diverse Temperature, pH, and UV Irradiation

**Figure 2:**
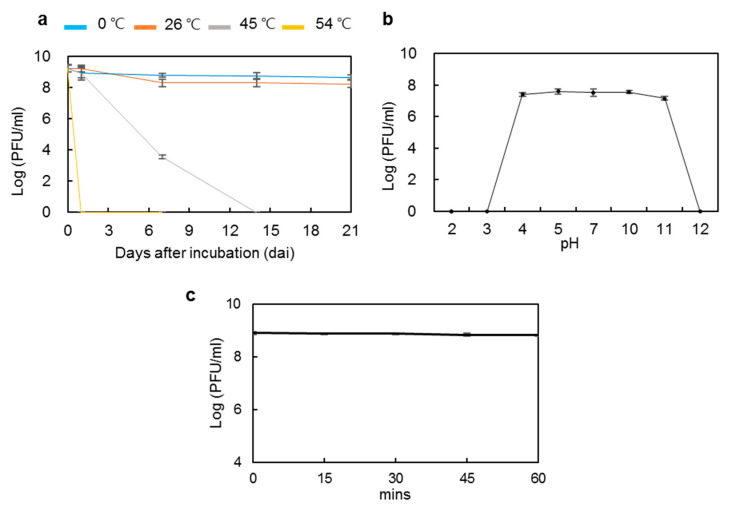
Stability of phiPccP-1 lytic activity under diverse temperatures, pH and UV light (λ = 365 nm) against *Pectobacterium odoriferum* Pco14. (**a**) phiPccP-1 lysate aliquots were incubated at different temperatures for 1 h. (**b**) phiPccP-1 lysate aliquot in buffers with various pH values were incubated overnight at 4 °C. (**c**) phiPccP-1 lysate aliquots were irradiated with UV light for 1 h. Then, remnant viable phage titers were determined by doting 10-fold dilution solution against *P. odoriferum* Pco14. Presented values are average of 3 replicates, and error bars indicate standard deviation.

Physico-chemical factors, such as temperature, pH, and sunlight, have been described to influence phage survival and persistence [52]. To assess the potential of phiPccP-1 for biocontrol of soft rot disease, its stability was investigated under various temperatures and pH conditions. The active phiPccP-1 titers were unchanged after three weeks of incubation at 0 °C, while a 10-fold decrease was observed after the first week at 26 °C, and then it was maintained at the same level during the last two weeks. However, at 45 °C, its lytic activity was reduced and completely lost 14 days after incubation (Figure 2a). At 54 °C, the lytic activity was completely lost even one day after incubation. These results indicated that phiPccP-1 bacteriophage can be stored at the refrigerator and room temperature, and importantly it can keep its lytic activity in field where Kimchi cabbage is cultivated in Korea because its optimal cultivation temperature in field is 18–20 °C.

The acidity and alkalinity of the environment are also important factors to influence phage stability. The results of the pH stability indicated that phiPccP-1 was very stable in the pH range of 4.0–11.0 and completely inactivated at extreme pH values of 2.0, 3.0 and 12.0 (Figure 2b). Among environmental factors, UV light was considered as the most destructive to biocontrol efficacy [53]. To check if the phage application is suitable under field conditions of South Korea, the stability of phiPccP-1 was examined for 1 h treatment under UV-A (λ = 365 nm). The concentration of phiPccP-1 bacteriophage was maintained as the initial concentration throughout the treatment period (Figure 2c). Altogether, these results demonstrated that phiPccP-1 bacteriophage can be efficiently survived in various environmental conditions, resulting in its potential for soft-rot disease control under the field conditions in Korea.

### 3.5. Analysis of phiPccP-1 Genome 

The bacteriophage phiPccP-1 genome was sequenced by PacBio RS II method. It is a linear DNA with 40,487 bp in length (GenBank accession number: MW001769) and G+C content of 47.78% and has a 209 bp of terminal repeats at its genomic termini. A total of 49 open reading frames (ORFs) were predicted and identified on the positive strand (Table S2), and no tRNA gene was found in the genome. Based on the analysis using a combination of BLASTP and InterProScan 5, putative functions of 26 out of the 49 predicted ORFs (53%) were assigned. These gene products can be categorized into DNA replication, virion structure, and host lysis function (Figure S2, Table S2). The observation of the putative RNA polymerase gene, as well as its genome organization, suggested that phiPccP-1 may belong to the described family *Autographivirinae* [54]. In addition, no antibiotic resistance gene and lysogen-related genes encoding proteins such as integrases, excisionase, repressors, and antirepressors were observed by genomic analysis, suggesting that this phage is lytic and suitable for phage therapy.

**Figure 3:**
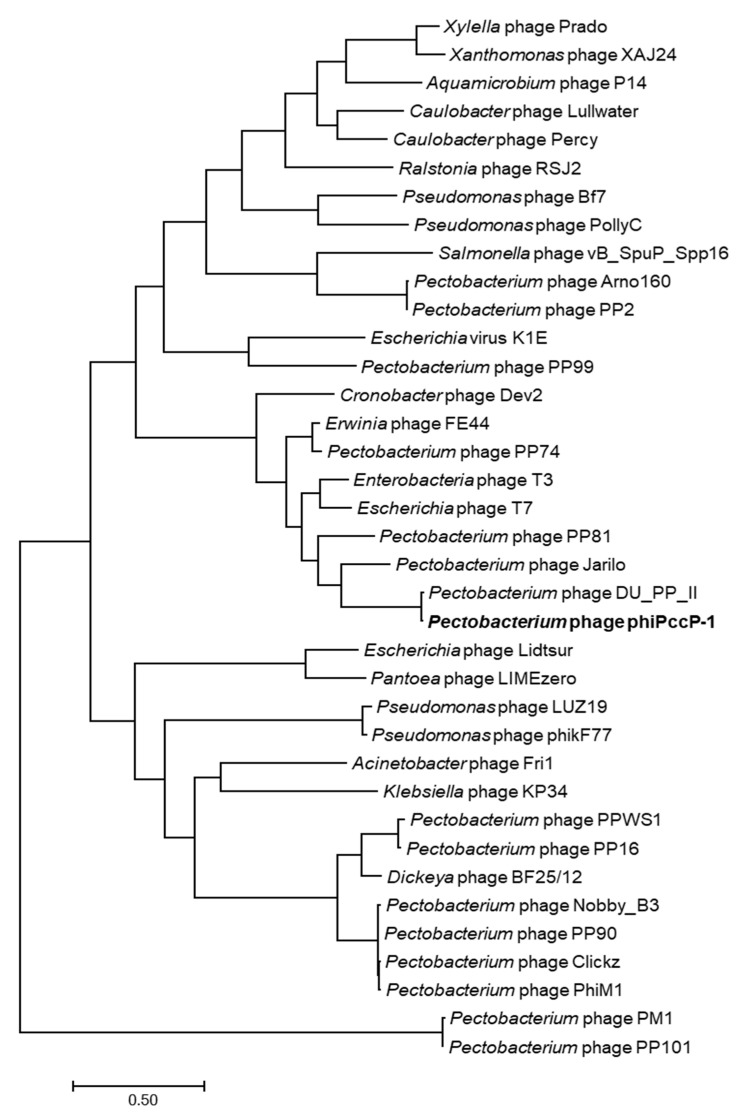
Phylogenetic tree of bacteriophage phiPccP-1 using concatenated amino acid sequences of phage DNA polymerase, DNA-directed RNA polymerase, and major capsid proteins aligning by MUSCLE in MEGA7. The phylogenetic tree was generated using the maximum-likelihood method with the Poisson correction model and one thousand bootstrap repetitions.

The phiPccP-1 genome showed the best match with the uncharacterized *Pectobacterium* phage DU_PP_II (GenBank accession number NC_047886.1, 98.2% identity, 99% coverage). The sequence comparison showed that the average nucleotide identity (ANI) between these two genomes was 97.28% (calculated by ANI, http://enve-omics.ce.gatech.edu/ani/, accessed on 1 March 2021). Both phage genomes showed similar genetic structures with a high level of identity (Figure S2). However, an additional 211 bp was observed at N terminal of PhiPccP-1 possessing the 209 bp of terminal repeat. Phylogenetic studies of the phiPccP-1 with amino acid sequences of DNA polymerase, DNA-directed RNA polymerase and major capsid proteins was employed to clarify the evolutionary origin of phiPccP-1. All these phylogenetic results clearly grouped phiPccP-1 together with phage DU_PP_II in a distinct branch. A phylogenetic analysis using concatenated amino acid sequences of these three proteins was conducted (Figure 3). The results demonstrated that phiPccP-1 belongs to the *Unyawovirus* genus, *Studiervirinae* subfamily of the *Autographivirinae* family.

### 3.6. Control Efficacy of phiPccP-1 Against Soft-Rot Disease in the Detached Mature Leaves of Kimchi Cabbage

**Figure 4:**
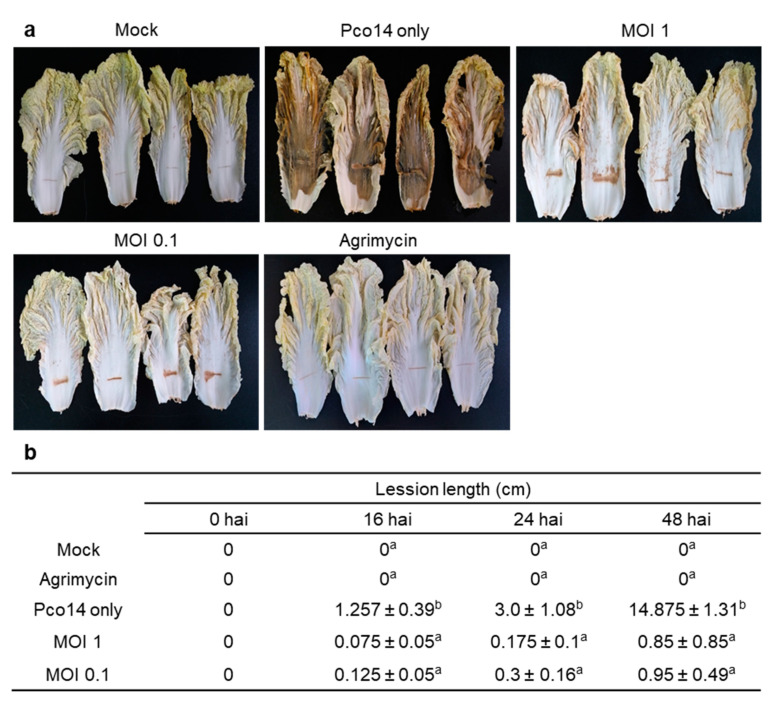
Prevention of soft rot disease pretreated with phiPccP-1 on Kimchi cabbage leaves. Mature Kimchi cabbage leaves were pretreated with phiPccP-1, Agrimycin^®^, or 10 mM MgCl_2_ as negative control by spraying. After 2 h, all the leaves were wounded using sterilized surgery blade dipping in 10^9^ CFU mL^−1^ *Pectobacterium odoriferum* Pco14 suspension. (**a**) Kimchi cabbage leaves observed after 48 h after pathogen inoculation (hai). (**b**) The lesion lengths in 4 leaves per treatment were measured at the indicated time points until 48 hai. The disease severity was presented by the average lesion length of 4 replicates and standard deviation. The different characters next to the disease severity indicate the statistical analysis results of Ducan’s multiple range test (*p* < 0.05) at each time point.

After harvest, bacterial soft rot easily develops within a few h in nearly all fresh vegetables, resulting in the quantity reduction and economic loss [26]. To investigate the efficacy of phiPccP-1 on protecting Kimchi cabbage after harvest, the commercially harvested ones were employed. The tissue maceration was firstly observed around the wounding sites in Pco14-inoculated leaves after 16 h, and symptom was progressed vertically and reached to both edges of the leaves at 48 h after pathogen inoculation (Figure 4a,b). Although the tissue maceration was also observed around the wounding sites in leaves pretreated with both multiplicity of infections (MOI 1 and 0.1) of phiPccP-1, it was only progressed approximately 1 cm vertically from the initial wounding sites in both treatments with different phage concentration (Figure 4a,b). Overall control efficacy of phiPccP-1 pretreatment was almost 95% (Figure 4b). No symptoms were observed in the leaves pretreated with antibiotic. These results imply that phiPccP-1 effectively protected mature Kimchi cabbage leaves after harvest from *Pectobacterium* pathogen. 

### 3.7. Control Efficacy of phiPccP-1 against Soft-Rot Disease in Kimchi Cabbage Seedlings

**Figure 5:**
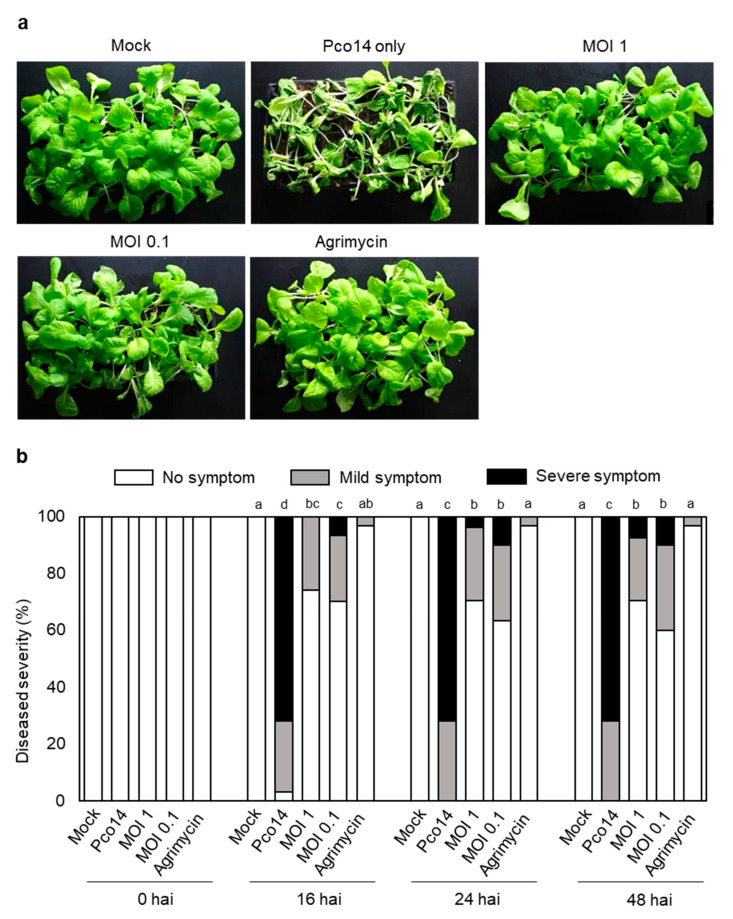
Prevention of soft-rot disease in Kimchi cabbage seedlings by phiPccP-1. Three-week-old Kimchi cabbage seedlings were pretreated with phiPccP-1 or Agrimycin® by spraying on the seedlings. After 2 h, all seedlings were sprayed with 10^8^ CFU mL^−1^ of *Pectobacterium odoriferum* Pco14. (**a**) Kimchi cabbage seedlings observed after 48 h after pathogen inoculation (hai). (**b**) Disease severity was analyzed to see control efficacy of bacteriophage in Kimchi cabbage. Disease severity was divided into three categories: no symptom, no visible symptom; mild symptom, mild maceration only on leaves; severe symptom, severe maceration spread over the leaves and stems or dead plants. Thirty seedlings were used for each treatment. Different letters indicated statistically significant difference among treatments at the same time point based on the nonparametric Kruskal–Wallis test and Tukey test (*p* < 0.05).

To determine both the prophylactic and therapeutic effect of phiPccP-1 in Kimchi cabbage seedlings, three-week-old seedlings were used. For the prophylactic treatment, seedlings were pretreated with two different MOIs (MOI 1 and 0.1) of phiPccP-1 and sprayed with Pco14 2 h after phage pretreatment. Approximately 96% of Pco14-treated seedlings showed the soft-rot symptoms 16 h after pathogen inoculation (hai), and all seedlings showed severe symptoms after 24 hai (Figure 5a,b). In contrast, the application of phiPccP-1 prior to the pathogen inoculation resulted in a decreased incidence of soft-rot disease: approximately 60% plants were still healthy after 48 hai, but less effective than with Agrimycin® treatment with only 6% plants showing mild symptoms. In addition, the treatment of MOI 0.1 showed the lower efficiency to control disease than the treatment of MOI 1.0 (Figure 5a,b). These results indicate that the pretreatment of phiPccP-1 can reduce symptom development more than 60% in Kimchi cabbage seedlings, which is consistent with results in the detached mature leaves. 

**Figure 6:**
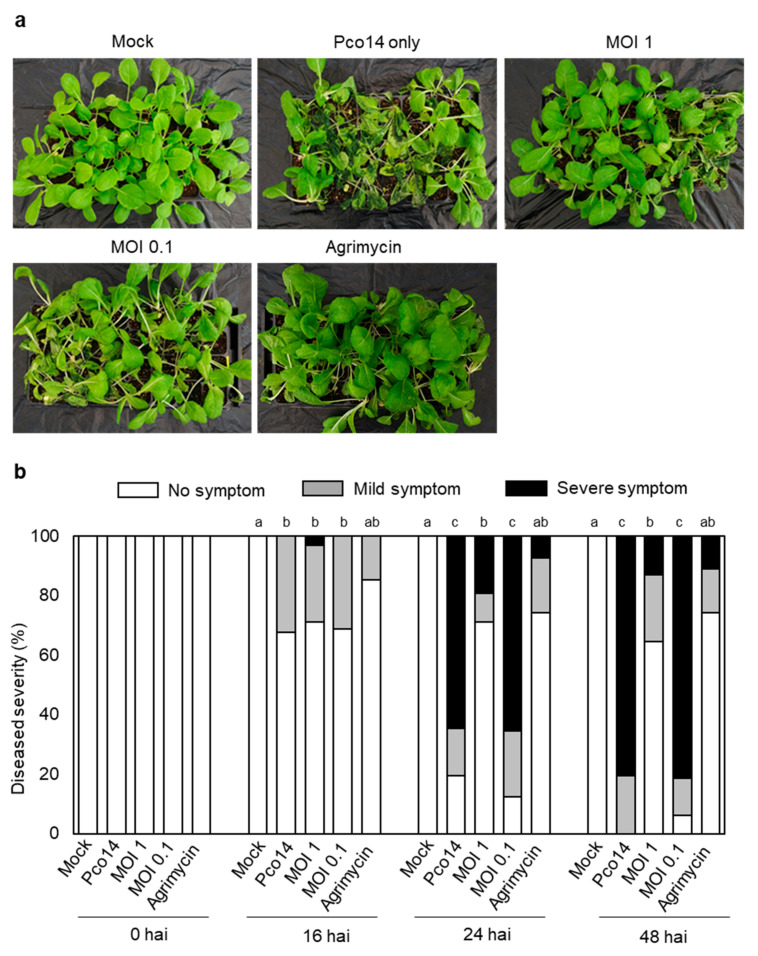
Therapy of soft-rot disease in Kimchi cabbage seedlings by phiPccP-1. Three-week-old Kimchi cabbage seedlings were pretreated with 10^8^ CFU mL^−1^ of *P*. *odoriferum* Pco14 by spraying on the seedlings. After 6 h, all the plants were sprayed with phiPccP-1 or Agrimycin^®^. (**a**) Kimchi cabbage seedlings observed after 48 h after pathogen inoculation (hai). (**b**) Disease severity was analyzed to see control efficacy of bacteriophage in Kimchi cabbage. Disease severity was divided into three categories: no symptom, no visible symptom; mild symptom, mild maceration only on leaves; severe symptom, severe maceration spread over the leaves and stems or dead plants. Thirty seedlings were used for each treatment. Different letters indicate statistically significant difference among treatments at the same time point based on the nonparametric Kruskal–Wallis test and Tukey test (*p* < 0.05).

For the therapeutic treatment, two MOIs (MOI 1 and 0.1) of phiPccP-1 were sprayed to seedlings at 6 hai. As the soft-rot control, the mild water-soaking areas were observed in Pco14-inoculated seedlings at 6 hai. Such symptoms were rapidly spread and reached 100% seedlings at 48 hai (Figure 6a,b). In contrast, almost 90% of plants, including 60% of healthy plants, were protected against Pco14 infection after 48 hai in the treatment of MOI 1, which is almost similar to antibiotic treatment (Figure 6b). However, only approximately 20% were observed in the treatment of MOI 0.1. These results indicate that foliar spraying with phiPccP-1 or Agrimycin® effectively reduced the disease incidence even after pathogen infection.

## 4. Discussion

Bacteriophages that attack different *Pectobacterium* species have been previously characterized [33,34,36,38,55]. However, no particular phage has been reported to be capable of infecting and killing *P. odoriferum*, since this species was reclassified from *P. carotovorum*. PhiPccP-1 is a bacteriophage carrying antibacterial activity against *P. odoriferum* as well as other two species, *P. carotovorum* and *P. brasilience*. These suggested that phiPccP-1 may be a potential biological agent to control soft-rot disease caused by not only *P. odoriferum*, but also two other important *Pectobacterium* species. PhiPccP-1 phage possesses similar morphological characteristics as well as genome organization to previous characterized *Pectobacterium* phages belonging to family *Autographivirina* (Figure 1b, Figure S2), and phylogenetic studies further grouped it to *Unyawovirus* genus, *Studiervirinae* subfamily.

The success of biocontrol of bacterial plant diseases with bacteriophages is influenced by several environmental factors such as pH, temperature, and sunlight irradiation [56]. PhiPccP-1 was stable in the pH range 4–11, which was similar to the *Podoviridae* phage PP1 [33]. In contrast to other reported *Pectobacterium* phages, phiPccP-1 was more sensitive to temperature. The antibacterial activity of phiPccP-1 was partially reduced and completely lost after overnight incubation at 45 °C and 54 °C, respectively. However, the activity retained after overnight incubation at 0 °C or room temperature. Both Kimchi cabbages and *Pectobacterium* spp. require relatively low temperature for optimum development (under 20 °C) and pathogenic activity [11,57,58,59]. Further, phiPccP-1 exhibited the stability of its antibacterial activity under UV-A treatment, which is considered as the most destructive and important factor for the success of phage application in field [53]. Therefore, phiPccP-1 looks still suitable for controlling bacterial soft rot disease in Kimchi cabbages in field. 

MOI value is a considerable parameter in phage application. Results from killing curve assay demonstrated that phiPccP-1 possibly suppressed the growth of *P. odoriferum* Pco14 and *P. carotovorum* Pcc15 in all tested MOIs. Generally, the higher MOI is treated, the faster reduction is observed in both bacterial strains. However, such faster reduction was observed only in the first 2 h and then maintained as similar level in all MOI treatments (Figure 1c,d). The rapid evolution of bacteriophage-resistant bacteria was also observed around 6 h of inoculation (Figure 1c,d). The frequency of BIMs development was monitored at 7 × 10^−7^. However, phiPccP-1 was still able to infect and kill the selected BIMs with lower efficiency. These findings support that less bacteriophage phiPccP-1 can be applied to control soft rot, and its effect can be sustained for a longer period after phage treatment.

Spray treatment of phiPccP-1 on Kimchi cabbage leaves significantly reduced the spreading of soft-rot disease caused by *P. odoriferum* Pco14 (Figure 4). This suggested that phiPccP-1 can be applied for protecting Kimchi cabbages against the soft-rot disease caused by *P. odoriferum* after harvest. Furthermore, the control efficacy was not much different between two MOI treatments. This trend was also observed in POP72 phage application to control the soft-rot disease caused by *P. carotovorum* Pcc27 [38]. Remarkably, the soft-rot symptom observing in treatment with only *P. odoriferum* Pco14 was more severe and quickly spread compared to those caused by *P. carotovorum* Pcc27 consistent with previous reports [21,23]. In addition, the prophylactic and therapeutic potential of phiPccP-1 against soft-rot disease caused by *P. odoriferum* Pco14 was demonstrated in the greenhouse. The difference in pathogenic ability and spreading rate between *P. carotovorum* and *P. odoriferum* once again demonstrated by the rapid invasion after 48 h of pathogen treatment (Figure 5 and Figure 6), compared to a previous report of *Pectobacterium* bacteriophage PP1 [33]. Consistently, the phiPccP-1 application using hand sprayer 2 h before or 6 h after *P. odoriferum* Pco14 successfully protected approximately 90% of seedlings from soft rot disease after 48 hai. All results indicated the significant potential of phiPccP-1 as a biocontrol agent for control of soft rot.

To fully explore the potential of lytic bacteriophage phiPccP-1 in practice, additional studies for optimizing its long-term effectiveness such as phage formulation and prophylactic and therapeutic efficacy in field are required. Furthermore, the isolation of other phages to form a phage mixture should be performed to overcome the limitation in target specificity as well as widening the host range covered.

## Data Availability

All data are contained within the article.

## Supplementary Materials

The following are available online at https://www.mdpi.com/article/10.3390/microorganisms9040779/s1, Figure S1: PhiPccP-1 lytic activity against the wild type and spontaneous mutants *P. odoriferum* Pco14, Figure S2: Genome alignment of bacteriophage phiPccP-1 with the closest phage DU_PP_II, Table S1: Host ranges of phiPccP-1, Table S2: List of ORFs and predicted functions in the complete genome of the bacteriophage phiPccP-1.
