# Supplementary material for: Biocontrol of Soft Rot Caused by Pectobacterium odoriferum with Bacteriophage phiPccP-1 in Kimchi Cabbage
Source: Microorganisms. 2021 Apr 8;9(4):779. doi: 10.3390/microorganisms9040779 (PMC8068257; doi:10.3390/microorganisms9040779)
Supplement: Supplementary file 1 [file microorganisms-09-00779-s001.pdf]

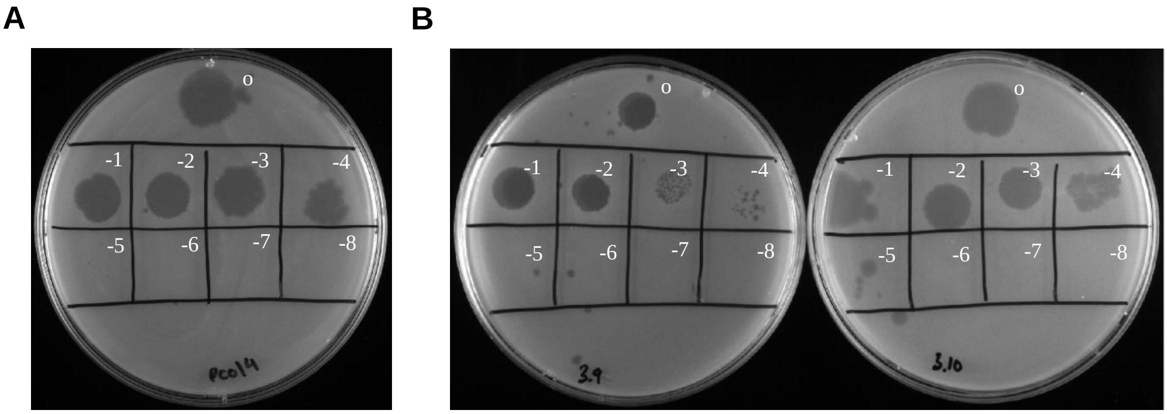

Figure S1. PhiPccP-1 lytic activity against the wild type and spontaneous mutants *P. odoriferum* Pco14. The crude phiPccP-1 lysate was diluted up to 10^8 times, and 10µL of each solution were spotted on lawn of *P. odoriferum* Pco14 (A) or its two selected spontaneous mutants (B). The phage activity was evaluated by comparing the characteristic of formatting plaques using the spotting assay. Numbers indicate $\log_{10}$(number).

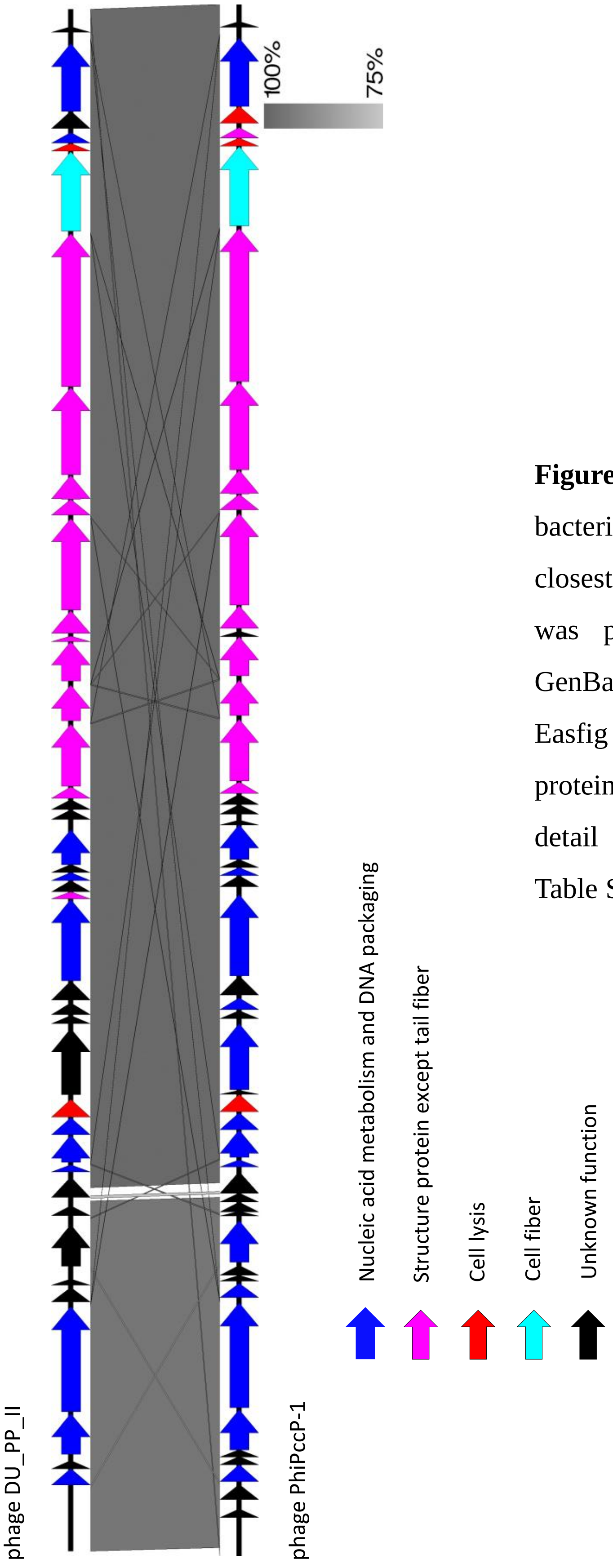

Figure S2. Genome alignment of bacteriophage phiPccP-1 with the closest phage DU_PP_II. The alignment was performed with BlastN at the GenBank database and visualized with Easfig 2.2.5 The putative gene encoding proteins were indicated via arrow. The detail information on each ORF is in Table S2.

Table S1. Host ranges of phiPccP-1.

Bacteria	Isolation sources	Isolated sites	Isolation year	Lytic activity*
P. odoriferum Pco14	Kimchi cabbage	Pyeongchang	1997	o
P. odoriferum	Lettuce	Pyeongchang	1997	o
P. odoriferum	Kimchi cabbage	Gangneung	2019	-
P. odoriferum	Kimchi cabbage	Gangneung	2019	o
P. odoriferum	Kimchi cabbage	Jeoseong	2019	o
P. odoriferum	Kimchi cabbage	Jeoseong	2019	o
P. odoriferum	Kimchi cabbage	Namyangju	1997	-
P. odoriferum	Carrot	Pyeongchang	1997	o
P. brasilience	Tomato	Namyangju	1997	o
P. brasilience	Potato	Chuncheon	1997	o
P. brasilience	Chickweed	Yangjae	2010	o
P. brasilience	Chrysanthemum	Yangjae	2010	-
P. brasilience	Eggplant	Jinju	2012	o
P. brasilience	Eggplant	Jinju	2012	o
P. carotovorum	Cucumber	Buyeo	1997	-
P. carotovorum	Potato	Jeju	1997	o

P. carotovorum	Kimchi cabbage	Hongcheon	1997	-
P. carotovorum	Kimchi cabbage	Hongcheon	1997	o
P. carotovorum Pcc15	Cabbage	Pyeongchang	1997	o
P. carotovorum	Oriental melon	Buyeo	1997	o
P. carotovorum	Tobaco	Yesan	1998	-
P. carotovorum	Tomato	Chungju	2000	o
P. carotovorum	Calla	Seoul	2012	-
P. carotovorum	Kimchi cabbage	Suwon	2019	-
P. carotovorum				o
P. carotovorum	Kiwi	Suncheon	2008	-
P. parmentieri				-
P. parmentieri	Potato	Jeju	1999	-
P. parmentieri	Potato	Jeju	1999	-
P. parmentieri				-
P. atrosepticum				-
P. atrosepticum				-
P. betavasculorum				-
P. wasabiae				-
P. versatile				o

Dickeya dadantii	Potato	Jeju	2000	-
Dickeya dadantii	Welsh onion	Suwon	2000	-
Dickeya fangzhongdai				-
Dickeya fangzhongdai	Moth orchid	Namyangju	2000	-

* Lytic activity of phiPccP-1 against tested bacterial strains was indicated by circle, while the dashes showed the negative results.

Table S2. List of ORFs and predicted functions in the complete genome of the bacteriophage phiPccP-1

Gene_ID	Start	End	aa	Predicted functions
phiPccP-1_01	999	1226	227	hypothetical protein
phiPccP-1_02	1467	1865	398	hypothetical protein
phiPccP-1_03	1938	2381	443	serine recombinase
phiPccP-1_04	2372	2587	215	hypothetical protein
phiPccP-1_05	2571	2765	194	hypothetical protein
phiPccP-1_06	2762	3808	1046	protein kinase
phiPccP-1_07	3859	6591	2732	RNA polymerase
phiPccP-1_08	6722	7096	374	hypothetical protein
phiPccP-1_09	7149	7310	161	hypothetical protein
phiPccP-1_10	7310	7567	257	hypothetical protein
phiPccP-1_11	7646	8728	1082	ATP-dependent DNA ligase
phiPccP-1_12	8847	8972	125	hypothetical protein

phiPccP-1_13	8956	9216	260	hypothetical protein
phiPccP-1_14	9213	9443	230	hypothetical protein
phiPccP-1_15	9440	10000	560	hypothetical protein
phiPccP-1_16	10124	10312	188	inhibitor of host bacterial RNA polymerase
phiPccP-1_17	10379	11089	710	putative single-stranded DNA-binding protein
phiPccP-1_18	11092	11541	449	Endonuclease
phiPccP-1_19	11559	12014	455	Endolysin
phiPccP-1_20	12019	12117	98	hypothetical protein
phiPccP-1_21	12134	13855	1721	DNA primase/helicase
phiPccP-1_22	13986	14198	212	hypothetical protein
phiPccP-1_23	14218	14529	311	hypothetical protein
phiPccP-1_24	14597	15100	503	hypothetical protein
phiPccP-1_25	15100	17214	2114	DNA polymerase
phiPccP-1_26	17419	17709	290	hypothetical protein

phiPccP-1_27	17709	17918	209	HNS binding protein
phiPccP-1_28	17918	18133	215	hypothetical protein
phiPccP-1_29	18126	19037	911	Exonuclease
phiPccP-1_30	19025	19153	128	hypothetical protein
phiPccP-1_31	19309	19557	248	hypothetical protein
phiPccP-1_32	19560	19826	266	hypothetical protein
phiPccP-1_33	19851	20150	299	tail assembly protein
phiPccP-1_34	20173	21783	1610	head-to-tail joining protein
phiPccP-1_35	21876	22799	923	capsid assembly protein
phiPccP-1_36	22909	23931	1022	capsid and scaffold protein
phiPccP-1_37	23931	24077	146	major capsid protein
phiPccP-1_38	24151	24741	590	tail tubular protein A
phiPccP-1_39	24756	27140	2384	tail tubular protein B
phiPccP-1_40	27232	27639	407	internal virion protein

phiPccP-1_41	27642	28265	623	capsid scaffolding protein
phiPccP-1_42	28277	30550	2273	internal virion protein C
phiPccP-1_43	30571	34557	3986	internal virion protein D
phiPccP-1_44	34622	36688	2066	tail fiber protein
phiPccP-1_45	36703	36909	206	Holin
phiPccP-1_46	36912	37187	275	DNA maturase A
phiPccP-1_47	37296	37745	449	hypothetical protein
phiPccP-1_48	37732	39510	1778	DNA packaging protein B
phiPccP-1_49	39795	39944	149	hypothetical protein
